# Activation of the 5-HT1A Receptor by Eltoprazine Restores Mitochondrial and Motor Deficits in a *Drosophila* Model of Fragile X Syndrome

**DOI:** 10.3390/ijms25168787

**Published:** 2024-08-13

**Authors:** Anna Vannelli, Vittoria Mariano, Claudia Bagni, Alexandros K. Kanellopoulos

**Affiliations:** 1 Department of Fundamental Neurosciences, University of Lausanne, 1005 Lausanne, Switzerland; 2 Department of Biomedicine and Prevention, University of Rome Tor Vergata, 00133 Rome, Italy

**Keywords:** intellectual disability, synaptic transmission, serotonin, neuromuscular junction, FXS therapy, eltoprazine

## Abstract

Neurons rely on mitochondrial energy metabolism for essential functions like neurogenesis, neurotransmission, and synaptic plasticity. Mitochondrial dysfunctions are associated with neurodevelopmental disorders including Fragile X syndrome (FXS), the most common cause of inherited intellectual disability, which also presents with motor skill deficits. However, the precise role of mitochondria in the pathophysiology of FXS remains largely unknown. Notably, previous studies have linked the serotonergic system and mitochondrial activity to FXS. Our study investigates the potential therapeutic role of serotonin receptor 1A (5-HT1A) in FXS. Using the *Drosophila* model of FXS, we demonstrated that treatment with eltoprazine, a 5-HT1A agonist, can ameliorate synaptic transmission, correct mitochondrial deficits, and ultimately improve motor behavior. While these findings suggest that the 5-HT1A-mitochondrial axis may be a promising therapeutic target, further investigation is needed in the context of FXS.

## 1. Introduction

The brain’s energy demand is high, requiring 20% of the body’s energy supply to maintain and support various processes involved in brain development and functions throughout an individual’s life, including neurogenesis, synaptic plasticity, and all types of behaviors coordinated by brain activity [1,2,3,4,5,6]. Mitochondria are the primary source of cellular energy production and reactive oxygen species (ROS) formation. They regulate calcium signaling and lipid and steroid metabolism, but, most importantly, they are involved in other cellular functions, such as proliferation, apoptosis, and autophagy [7,8,9,10,11,12]. In highly polarized cells like neurons, mitochondria are also present at the synapses to supply energy for synaptic function and balance Ca^2+^ signals to coordinate synaptic communication. For example, mitochondria in the pre- and post-synaptic compartments are essential for maintaining synaptic activity, producing ATP through oxygen and glucose [3] that can, in part, be used for processes like local protein synthesis [13,14], endocytosis, exocytosis [15], and cytoskeleton remodeling [16]. Alterations in mitochondrial function and morphology could contribute to Fragile X Syndrome (FXS) neuropathology by impairing oxygen supply. Consistently, alterations in mitochondrial homeostasis have been associated with several brain diseases, such as Parkinson’s disease (PD) and Alzheimer’s disease (AD) [7,17], but also with neurodevelopmental disorders such as Autism Spectrum Disorders (ASD), epilepsy, and schizophrenia [18,19,20,21,22,23,24,25,26,27]. Understanding mitochondrial pathogenesis in diseases and applying interventions requires knowing how mitochondrial homeostasis is regulated.

This study aims to address this question by taking advantage of the well-established *Drosophila* model for FXS [23,28,29]. Despite its simplicity, *Drosophila* exhibits a wide range of complex behaviors and has well-defined physiological systems, such as a nervous system, cardiovascular system, and digestive system. This allows for comprehensive studies of its development, neurobiology, behavior, and disease status. Therefore, genetic or pharmacological interventions using this model could highlight new therapeutical avenues.

Fragile X Syndrome (FXS) is the most common monogenic cause of ASD [30,31], which is caused by the absence or mutations of Fragile Ribonucleoprotein 1 (FMRP), an RNA-binding protein that acts primarily as a regulator of local protein synthesis at synapses [32]. Clinically, FXS patients present a wide spectrum of symptoms such as developmental delay, hyperactivity, disrupted sleep, social impairments, and intellectual disability [33,34]. Furthermore, post-mortem brain studies using Golgi staining have revealed dendritic spine structural anomalies, suggesting that FMRP regulates synaptic development and plasticity [35]. Using different animal models, independent studies have shown that FMRP plays a role in synaptic transmission and plasticity [36].

*dfmr1* is the single homolog of the human gene in the *Drosophila* genome; *dfmr1^Δ50^* mutants, at both larval and adult stages, present altered locomotor activity associated with altered morphology of the neuromuscular junction (NMJ) and abnormal synaptic transmission [28,37,38,39]. In addition, lack of FMRP leads to an increased number and altered size and distribution of mitochondria at the NMJ level, impairments in mitochondrial plasticity (their ability to dynamically adapt their structure, function, and metabolism in response to various physiological and environmental cues), and polarity in dendrites and axons, respectively, along with elevated mitochondrial membrane proton leak, leading to increased metabolism and changes in protein synthesis [40,41,42,43,44].

In addition to the key role played by the mitochondria, several monoamines regulate NMJ function [45,46,47,48,49,50,51]. For example, serotonin levels were found to be dysregulated in a mouse model for FXS, namely the *Fmr1* KO mouse [52]. The selective activation of the 5-HT receptor was shown to ameliorate behavioral deficits such as hyperactivity, abnormal sensorimotor gating, and cognitive impairment. It is well established that modulation of the serotonergic system influences locomotion [47,48,53]. Furthermore, it was shown that *dfmr1^Δ50^* mutant flies present altered levels of serotonin in the brain [54]. 5-HT is a monoamine with a regulatory effect on locomotion and synaptic activity in flies [47,48,55]. Moreover, 5-HT plays an essential role in mitochondrial biogenesis in neurons and other cell types [56] through the mitochondrial master regulator PGC-1α [57,58,59]. These results suggest that the 5-HT–mitochondrial axis dysregulation in FXS could contribute to some of the observed deficits in neuronal plasticity and behavior. The critical role of serotonin in numerous brain functions and its involvement in various neurological and psychiatric disorders make it a significant target for drug development. Here, we assessed the effects of eltoprazine, a 5-HT1A agonist, in the *Drosophila* model of FXS on behavior, neuronal activity, and neuronal morphology and show how the serotonin pathways affect mitochondrial homeostasis.

## 2. Results

### 2.1. dfmr1^Δ50^ Mutants Have Locomotion Deficits That Are Rescued upon Eltoprazine Treatment

Here we investigated if eltoprazine, an agonist of the 5-HT1A receptor, could ameliorate the deficits in locomotor activity of the *dfmr1^Δ50^* mutant larvae, a behavioral feature that was previously reported [60]. 

**Figure 1:**
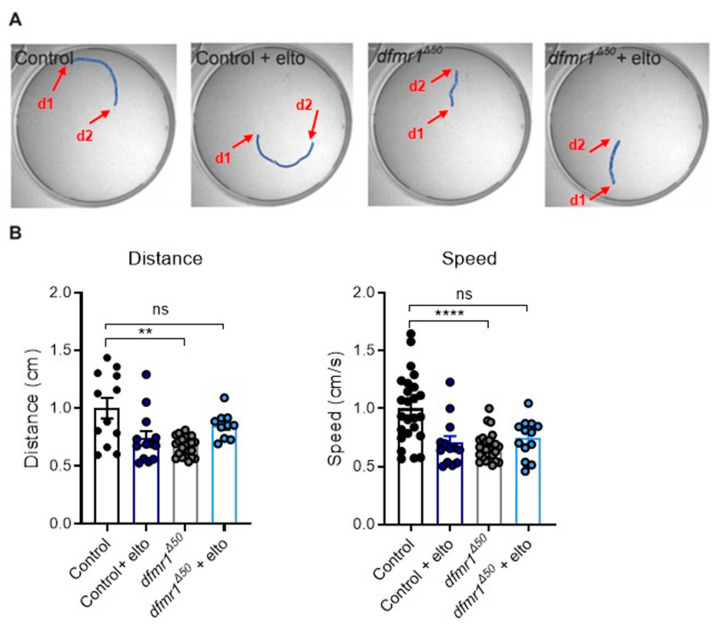
Activation of the 5-HT1A receptor by eltoprazine rescues motor behavior in *dfmr1^Δ50^* larvae. (**A**) Representative images of larval tracking on agarose plate for the various conditions tested. The red arrows indicate the distance that the larvae covered between points d1 and d2. (**B**) Quantification of the locomotor behavior for the control, *dfmr1^Δ50^* mutants, the control treated with eltoprazine, and *dfmr1^Δ50^* mutants treated with eltoprazine (elto). The analysis was performed automatically with Ethovision by measuring the distance and speed of the larvae. Each dot represents a single larva. Left panel: distance traveled by the larvae during a 1 min observation period. Right panel: crawling speed, measured as distance moved in cm per second. For each condition, *n* ≥ 15 single larvae. Data are shown as dot plots, and error bars represent the standard error of the mean; 2-way ANOVA, multiple comparisons: ** *p* < 0.01, **** *p* < 0.001, ns means no significant.

First, we analyzed distance and speed in freely moving crawling larvae as described in [60]. Consistent with previous observations [38,61], the *dfmr1^Δ50^* mutant larvae are less active than the controls (Figure 1A,B). Feeding of *dfmr1^Δ50^* mutant larvae with 1 mM of eltoprazine for 30 min increased the locomotor activity of the mutants compared to the controls (*w1118*). Of note, the same treatment on the control larvae significantly reduced their locomotion (Figure 1A,B).

### 2.2. Eltoprazine Restores the Synaptic Transmission at the NMJ of dfmr1^Δ50^ Mutants

**Figure 2:**
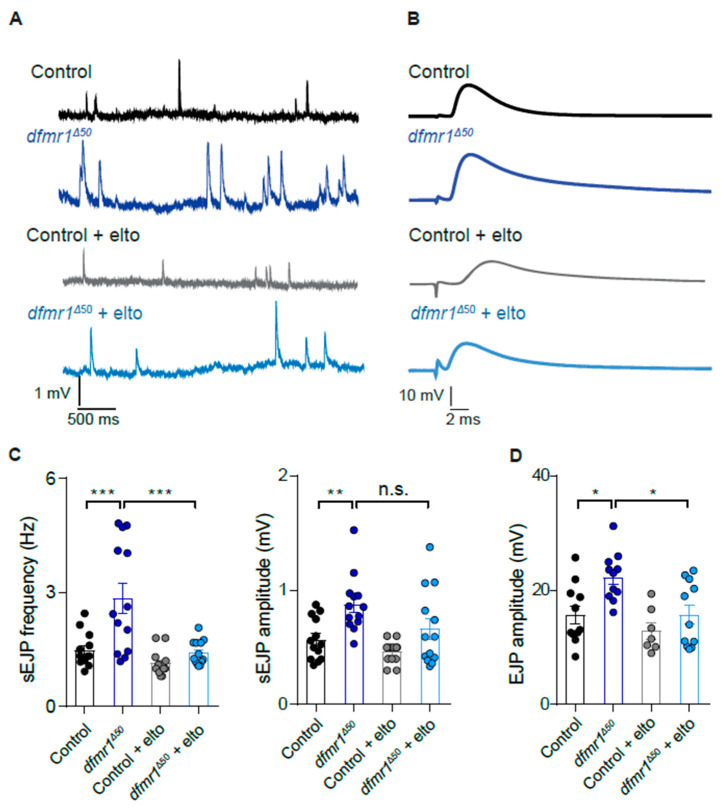
Activation of the 5-HT1A receptor ameliorates NMJ synaptic transmission of the *dfmr1^Δ50^* larvae. Representative traces of spontaneous (**A**) and evoked (**B**) activity in larvae from the control, *dfmr1^Δ50^,* the control treated with eltoprazine, and *dfmr1^Δ50^* treated with eltoprazine. (**C**) Quantification of spontaneous event frequency (Hz) (left) and amplitude (mV) (right). Each dot represents the measurement of a single NMJ. (**D**) Quantification of evoked EJP amplitude (mV). *n* ≥ 7 larvae for each condition. All the data are presented as dot plots, and error bars show the standard error of the mean. 2-way ANOVA, multiple comparisons: * *p* < 0.05, ** *p* < 0.01, *** *p* < 0.001, n.s. means no significant.

Locomotor defects in *dfmr1^Δ50^* larvae have been associated with altered synaptic function in the neuromuscular junction (NMJ) [28,62]. To explore a potential therapeutical approach, we measured the amplitude and frequency of the spontaneous Excitatory Junctional Potential (sEJP) in both control and *dfmr1^Δ50^* mutant larvae upon eltoprazine treatment. We show that the *dfmr1^Δ50^* mutant exhibits an increased amplitude and frequency of sEJP, and, upon electric stimulation of the NMJ, increased EJP amplitude in comparison with control flies (Figure 2A,C and Figure 2B,D, respective). It is known that 5-HT can directly modulate synaptic transmission at the NMJ [47,48]. Therefore, to test if the modulation of the 5-HT1A receptor can modulate NMJ transmission in the *dfmr1^Δ50^* mutant larvae, we applied eltoprazine acutely to the tissue. Recordings of spontaneous synaptic transmission in the presence of the drug revealed a significant amelioration of the sEJP frequency and partial rescue of the sEJP amplitude in the *dfmr1^Δ50^* mutant larvae (Figure 2C). Moreover, treatment with eltoprazine normalized the evoked EJP of the *dfmr1^Δ50^* mutant larvae to control levels (Figure 2D). These findings indicate that increased synaptic transmission could contribute to reduced larvae locomotion due to improper activation of the muscles and, consequently, uncoordinated muscle contractions. Restoration of synaptic activity with eltoprazine most probably acts by modulating the motoneuronal response to reduce the exaggerated release of glutamate.

### 2.3. Eltoprazine Restores Mitochondrial Dysregulations in dfmr1^Δ50^ Mutants

**Figure 3:**
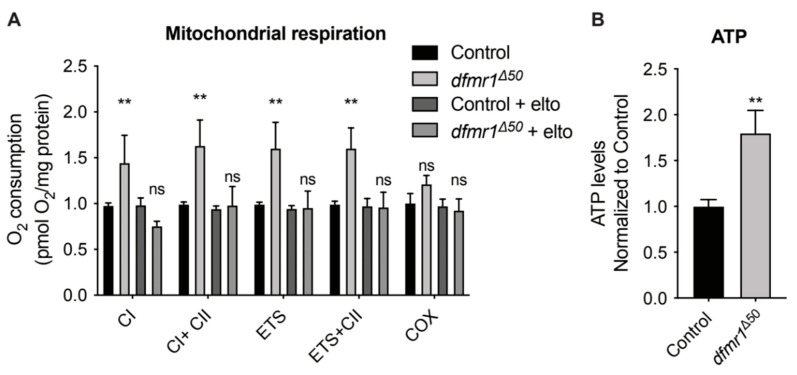
Eltoprazine treatment rescues NMJ mitochondrial hyperactivity in *dfmr1^Δ50^* larvae. (**A**) Quantification of oxygen consumption normalized per protein content and to the control. *n* ≥ 4 independent experiments per genotype (each with 3 NMJs), mean ± standard error of the mean. ** *p* < 0.01, ns means no significant, multiple *t*-test, corrected for multiple comparisons using the Sidak–Bonferroni method. (**B**) Quantification of ATP production levels in control and *dfmr1^Δ50^* larvae NMJs. Data represent *n* ≥ 4 independent experiments per genotype (each with 3 NMJs), expressed as mean ± standard error of the mean. Statistical significance was determined by *t*-tests (** *p* < 0.01) comparing the ATP levels of *dfmr1* mutant flies to those of wild-type control flies.

To investigate mitochondrial activity in *dfmr1^Δ50^* mutants based on FMRP regulation of mitochondrial function, morphology, and distribution in mammals [40,41], we measured oxygen consumption in the NMJs as previously described in [23]. We found that *dfmr1^Δ50^* mutants exhibit increased Complex I, Complex II, and electron transport of mitochondrial Oxidative Phosphorylation (OXPHOS) compared to control larvae (Figure 3A). Markedly, eltoprazine treatment normalized the increased mitochondrial activity in *dfmr1^Δ50^* mutant larvae (Figure 3A).

In addition, we analyzed the energy status of the NMJ and found that adenosine triphosphate (ATP) production is increased in *dfmr1^Δ50^* mutant larvae (Figure 3B).

**Figure 4:**
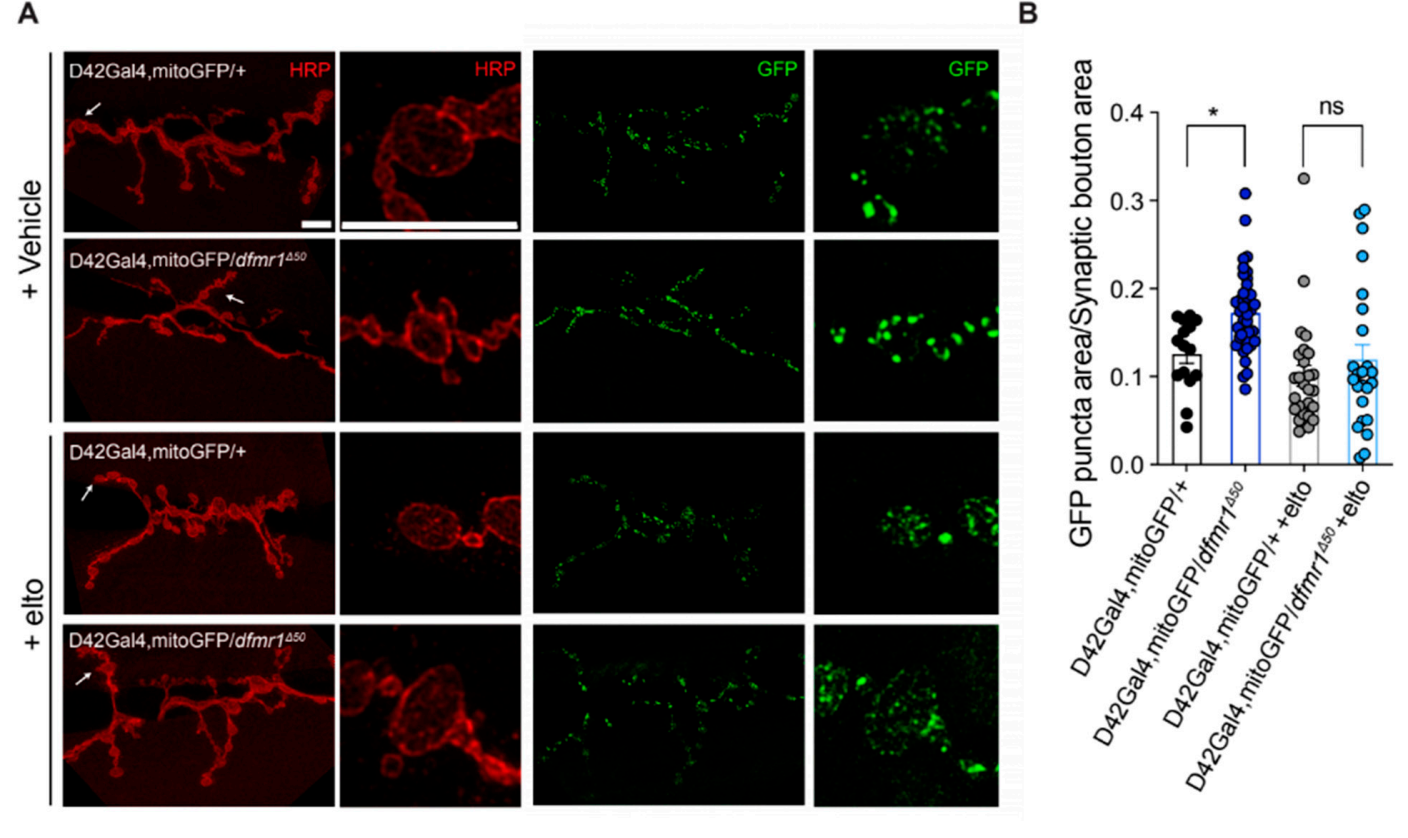
Eltoprazine restores mitochondrial puncta and distribution in the NMJ of *dfmr1^Δ50^* larvae. (**A**) Representative images of the NMJ (neuromuscular junction) synaptic boutons expressing mito-GFP (mitochondrial-targeted green fluorescent protein) and stained with a-HRP (anti-Horseradish Peroxidase). To visualize synaptic boutons at the *Drosophila* NMJ, a TRITC-HRP conjugated antibody was used, which provided visualization of the presynaptic terminals, facilitating the clear observation of their structure and organization. Arrows indicate the close-up magnification of axonal processes. Scale bar 10 um for all images. (**B**) Quantification of the mito-GFP puncta normalized to the synaptic boutons’ area and number. Each dot represents a single NMJ where at least 3 branches and 5 boutons for each branch were analyzed. *n* ≥ 15 NMJs. All data are shown as dot plots ± standard error of the mean; 2-way ANOVA (two-way analysis of variance), multiple comparisons, * *p* < 0.05, ns means no significant.

To determine if the increased mitochondrial activity was generated by increased mitochondrial mass, we measured the number and distribution of mitochondria at the synapses of motor neurons. Using the UAS/GAL4 system, we expressed mito-GFP (UAS) under the control of the motor neuron-specific D42 promoter (GAL4) which allows its expression in the motor neurons of the *dfmr1^Δ50^* mutant and control larvae (Figure 4A). Notably, *dfmr1^Δ50^* mutant larvae show increased expression of mito-GFP compared to controls, suggesting an increased number of mitochondria at the synapses of the NMJ (Figure 4A,B). Importantly, treatment with eltoprazine yielded a significant improvement in the quantity of mitochondria at the NMJ (Figure 4A,B). This observation strongly aligns with recent research underscoring the role of serotonin in regulating mitochondrial biogenesis [57,59,63] and supports the hypothesis that a dysregulation of energy metabolism in the NMJ of *dfmr1^Δ50^* mutant larvae may underlie the NMJ synaptic transmission [64,65] and motor impairments in FXS.

### 2.4. Mitochondrial Biogenesis Regulates Locomotor Behavior in dfmr1^Δ50^ Mutants

**Figure 5:**
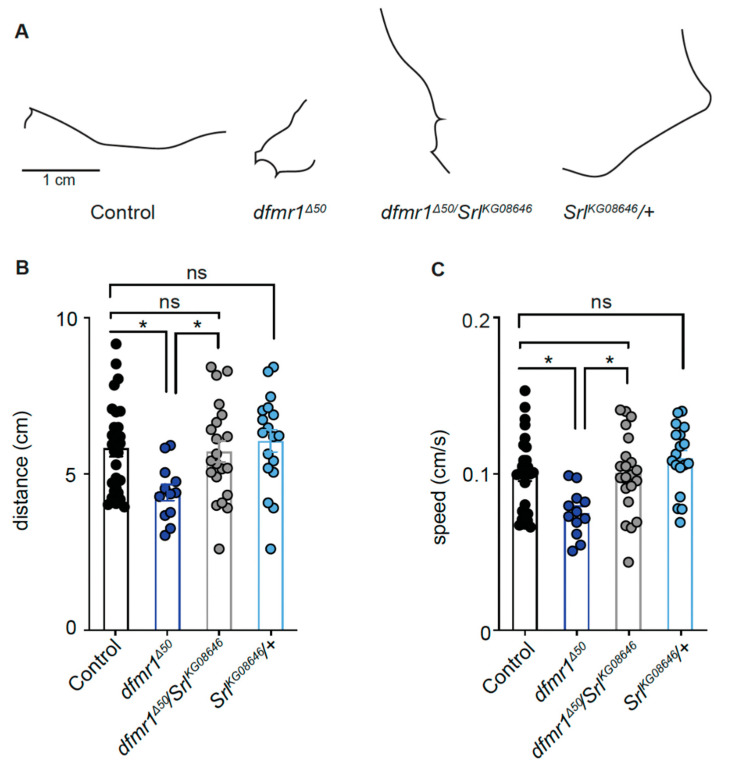
Genetic modulation of mitochondrial biogenesis marker PGC1A rescues motor behavior in *dfmr1^Δ50^* larvae. (**A**) Images representing larval tracking across various conditions. (**B**) Measurement of larval movement distance observed over 1 min. (**C**) Quantification of the crawling speed, measured as distance moved in cm per second. For each condition, *n* ≥ 7 single larvae. Data are shown as dot plots, and error bars represent the standard error of the mean; Kruskal–Wallis test followed by Dunn’s multiple comparisons test, * *p* < 0.05, ns means no significant.

The increased mitochondrial density and activity in *dfmr1^Δ50^* larvae may result from higher mitochondrial biogenesis, potentially regulated by serotonin through PGC1α, which is a target of FMRP [66]. Therefore, we hypothesize that the *dfmr1^Δ50^* larvae have a dysregulation of the serotonin–PGC1α–mitochondrial axis, ultimately affecting motor behavior in this FXS model. To test this hypothesis, we crossed a *Drosophila* mutant of the homolog of the mammalian *PGC1α* [67] called *Spargel* (*Srl^KG08646^*) expressing a hypofunctional allele with the *dfmr1^Δ50^* and assessed its locomotor behavior. This double mutant does not exhibit differences in motor behavior compared to control flies (Figure 5A,B). These findings further validate the dysregulation of the serotonin–mitochondrial axis in the NMJ of *dfmr1* mutant flies and elucidate the mechanistic effect of eltoprazine.

## 3. Discussion

Over the past three decades, numerous studies have investigated the pathophysiology of Fragile X syndrome, the most prevalent form of intellectual disability and a monogenic cause of autism [68]. The discovery of many different mechanisms working in neuronal and non-neuronal cells, at synapses, and in the cell body since the human gene *FMR1* was identified [69] has highlighted that multiple pathways are affected in FXS. Those identified mechanisms have propelled major efforts leading to more than 50 clinical trials, which unfortunately have not been very successful (clinicaltrials.gov).

Several pharmacological approaches have been working to tackle the metabotropic glutamate receptors (mGluRs) and the GABAergic system [70], which are considered leading players in FXS pathophysiology [38,71]. However, the unsuccessful clinical trials have highlighted the need to identify new molecules that could be used as targeted therapeutic approaches for specific clinical and behavioral phenotypes.

One promising target is the serotonergic system, which has been implicated in FXS and other neurodevelopmental disorders like autism spectrum disorder (ASD) and Rett syndrome [72,73,74]. Specifically, the 5-HT7A receptor has shown potential as a therapeutic target, with selective activation improving behavioral deficits in FXS mouse models [53,75]. Additionally, the 5-HT1A receptor, which modulates neuronal firing rates, has emerged as a potential target for treating motor deficits in FXS, given its association with neurodevelopmental disorders [53,54,76].

Previous studies demonstrate that eltoprazine, an agonist of the 5-HT1A receptor, can ameliorate dyskinetic movements in parkinsonian rats and monkeys and clinical studies [76,77,78,79,80]. A delay in motor development and problems in motor balance are often among the first notable signs of atypical development in children with FXS or ASD [81]. Specifically, it has been reported that an altered gait pattern associated with abnormal muscle activity in FXS subjects reduced knee and excessive hip and ankle flexion [82]. Hence, targeting 5-HT1A could potentially address certain motor deficits in FXS.

The *Drosophila* neuromuscular junction (NMJ) is an effective model system for studying synaptic development and function, giving us the advantage of translating physiological observations into behavioral phenotypes such as locomotion.

Consistent with previous findings, our data demonstrate that *dfmr1^Δ50^* mutants show decreased locomotion compared to controls [28,83,84]. Most importantly, upon eltoprazine treatment, the motor deficits of the *dfmr1^Δ50^* mutants were restored, indicating a role of 5-HT1A in modulating motor behavior (Figure 1). Serotonin modulates different aspects of locomotion, like forward locomotion and turning behavior in larvae [47,48,55], which is in line with our findings.

Locomotor behavior is considered a functional readout of synaptic transmission at the NMJ level, which was altered in *dfmr1^Δ50^* larvae [28,83,84]. Intracellular recordings of the NMJ revealed that the frequency and amplitude of the sEJP are increased compared to the controls, indicating dysfunction in both the pre-synaptic and post-synaptic components of the NMJ. Previously, this abnormal synaptic transmission was linked to altered subunit composition of glutamate receptors (GluRs) expressed in the muscles [28,62]. Here, we restored the synaptic transmission upon eltoprazine treatment (Figure 2). The rescue of the frequency of spontaneous activity is generally associated with presynaptic release, which is most probably acting on the soma of the motoneuron upstream of the GluRs. However, we noted only a partial restoration of the sEJP amplitude. This suggests that while this pathway can enhance the function of glutamate receptors, which are predominantly expressed at the *Drosophila* NMJ, it does not fully address all the synaptic deficits present in the FXS genetic model. This incomplete recovery likely stems from intrinsic defects in the glutamate receptors themselves, additional imbalances in other neurotransmitter systems, or broader abnormalities in synaptic architecture. These findings underscore the multifaceted nature of FXS pathology and highlight the need for a combination of therapeutic strategies to achieve a more comprehensive rescue of synaptic function.

Next, we attempted an initial characterization of the molecular mechanism downstream of the 5-HT1A receptor activity and its modulation by eltoprazine. It was reported that, in a mammalian system, the serotonergic system can modulate not only mitochondrial motility along the axons [85], but also mitochondrial biogenesis (MB) in cortical neurons, through the SIRT1-PGC1α axis [57,86]. In addition, mitochondria play an essential role in the modulation of synaptic transmission in neurons through ATP production and Ca^2+^ buffering and modulate the mobility of synaptic vesicles and neurotransmitter release [1,87,88]. By expressing mito-GFP in the punctal area (the synaptic level of the NMJ), we found not only an increased mitochondrial population and altered distribution (Figure 4), but also increased mitochondrial activity in *dfmr1^Δ50^* larvae in comparison to the controls (Figure 3). Of note, upon eltoprazine treatment, these phenotypes were comparable to control conditions. 

In recent years, mitochondria have emerged as significant contributors to FXS. Loss of FMRP in a mouse model for FXS results in impaired synaptic maturation associated with deficits in mitochondrial fusion [89] and increased mitochondrial activity in the cerebral cortex with preserved ATP production [90]. Moreover, ATP synthase c-subunit leakage in Fragile X is associated with synaptic morphology and behavioral deficits [43].

The increased mitochondrial distribution and activity suggest that the *dfmr1* mutants might present with an increased mitochondrial biogenesis, leading to altered motor behavior. In mammals, PGC1 (with three isoforms, PGC1α, PGC1β, and PRC) is a major transcription factor that induces the expression of genes encoding for mitochondrial proteins and regulates mitochondrial biogenesis [67]. The generation of double heterozygous mutants for *Spargel*, the single homolog of PGC1, and *Fmr1* genes shows normal locomotion comparable to control levels (Figure 5). This finding further supports our hypothesis that increased mitochondrial biogenesis leads to heightened mitochondrial activity, resulting in altered synaptic transmission at the NMJ and ultimately contributing to motor deficits in *dfmr1* mutant larvae. Of note, *PGC1α* mRNA has been reported to be a target of FMRP [91,92], therefore the observed alteration may be attributed to excessive translation of this mRNA, which is consistent with FMRP role as a repressor.

In conclusion, we propose a mechanism wherein the activation of the 5-HT1A receptor by eltoprazine modulates mitochondrial biogenesis and activity. By stabilizing mitochondrial function, it reduces neurotransmitter release and synaptic transmission, thereby facilitating proper motor behavior in *dfmr1^Δ50^* larvae. Our study indicates that targeting the 5-HT1A receptor–mitochondrial axis holds promise as a therapeutic approach for alleviating motor deficits in FXS. However, additional research is necessary to assess its viability for human therapeutic intervention.

## 4. Materials and Methods

### 4.1. Drosophila Stocks

Homozygous *dfmr1* mutant flies, *dfmr1^Δ50^* (Bloomington *Drosophila* Stock Center, #6930), were used in our study [28]. Flies were cultured in vials containing a standard *Drosophila* medium at 25 °C with 60–80% humidity in a 12 h light/dark cycle. The Canton-S *w^1118^* (iso1CJ) wild-type line served as a control. The *dfmr1^Δ50^* mutant flies were isogenized for 6 generations with the Cantonized w^1118^ background. The D42-Gal4 and UAS-mito-GFP flies were kindly provided by Prof. Patrik Verstreken (VIB/KULeuven). Briefly, The UAS-GAL4 system in *Drosophila* melanogaster enables precise control of gene expression. This system uses two components: the GAL4 gene, which encodes a yeast-derived transcriptional activator, and the UAS (Upstream Activating Sequence). Transgenic flies are created with GAL4 under a tissue-specific or inducible promoter. When these flies are crossed with flies carrying a UAS-linked gene of interest, GAL4 binds to UAS, activating gene transcription. This method allows for spatial and temporal control of gene expression, facilitating studies on gene function, development, and disease models [93]. The *Spargel* (*Srl*) mutant flies were a kind gift from Prof. Hugo Stocker (ETH Zurich). Third-instar larvae were used for all the experiments.

### 4.2. Larval Collection and Treatment

Third-instar larvae were collected by applying a solution of 20% sucrose on top of the food. The collected larvae were rinsed 3 times with 1X PBS at room temperature. Then, the larvae were exposed to eltoprazine hydrochloride (Santa Cruz Biotechnology, Dallas, TX, USA, cat. 98224-03-4) at a final concentration of 1 mM diluted in 5% sucrose solution for at least 30 min as described before [94]. Control larvae were treated with the vehicle in the same solution simultaneously.

For the electrophysiological experiments, a single larva was collected, and a neuromuscular junction (NMJ) fillet was prepared. After the NMJ preparation, the larva was exposed for 30 min to a modified minimal hemolymph-like solution, HL3.1 [95], containing 10 μM of eltoprazine or vehicle before the recording. 

### 4.3. Larval Crawling Behavior

The larval crawling behavior assay was performed as described before [60] with minor modifications. Briefly, a single larva was gently placed in the middle of a 10 cm arena which had been previously filled with 2% agar solution. The larva was allowed to acclimate for 30 s and then recorded with a Basler camera (Basler AG, Ahrensburg, Germany) for 1 min. Speed and distance were acquired and analyzed with EthoVision XT 13 (Noldus, Wageningen, The Netherlands). 

### 4.4. Electrophysiology

Intracellular NMJ recording from third-instar larvae was performed on muscles 5 and 6 in the abdominal segments 2/3/4. The recordings were made at room temperature with sharp glass electrodes filled with 3 M KCl. The nerves were stimulated by a brief (0.5–0.8 ms at 1 Hz) positive current via a suction electrode. The recording bath solution (HL3.1) had the following composition: 110 mM NaCl, 5 mM KCl, 10 mM NaHCO_3_, 10 mM MgCl_2_, 30 mM sucrose, 5 mM Trehalose, 5 mM HEPES (pH 7.2), and 1 mM CaCl_2_. A total of 60 responses were recorded per NMJ and averaged to give each datum. Miniature excitatory junctional potential (mEJP) was recorded for 1–2 min. The recordings were acquired with Clampex 10.7. (Molecular Devices, San Jose, CA, USA). The data were extracted with Clampfit software 10.7 (Molecular Devices, San Jose, CA, USA) and then analyzed with GraphPad Prism 7.03 (Boston, MA, USA).

### 4.5. Immunohistochemistry, Confocal Microscopy, and Image Analysis

Third-instar larvae carrying the UAS-mito-GFP marker driven by the motor neuron-specific D42-Gal4 driver were quickly dissected in 1X PBS and fixed in 4% formaldehyde for 20 min. Larvae were washed with 1X PBS containing 0.1% Triton X-100 (PBT) and blocked in 10% normal goat serum (NGS) (Sigma-Aldrich, Burlington, MA 01803, USA, cat. G9023) in PBT for 1 h, followed by overnight incubation with primary antibody in 5% NGS in PBT: Rhodamine (TRITC)-conjugated anti-horseradish peroxidase (HRP) (1:500, Jackson ImmunoResearch, West Grove, PA, USA). This dual-conjugated antibody combines the anti-horseradish peroxidase (HRP), that labels neuronal membranes in *Drosophila* with the tetramethylrhodamine isothiocyanate (TRITC) allowing the direct visualization of the presynaptic terminals of the NMJ. The TRITC component provided a distinct fluorescent signal, facilitating the observation of bouton morphology and distribution under a fluorescence microscope. After the washes in PBT, samples were mounted in Mowiol 4-88 mounting medium and imaged with a Leica SP8 confocal microscope in high-resolution mode (Hyvolution module from Leica) using a 60× oil immersion objective. Pictures were then deconvolved using Huygens 2 software (Scientific Volume Imaging B.V., Hilversum, The Netherlands).

All images analyzed were complete Z-stacks through NMJ 6/7 of abdominal segments A3 and A4. For mitochondrial density analysis, the sum area of the GFP+ puncta (UAS-mito-GFP) inside the synaptic bouton delineated by the TRITC-HRP staining was divided by the area of the synaptic bouton measured by the TRITC-HRP staining.

### 4.6. Mitochondrial Function Assays

High-resolution respirometry (OROBOROS Oxygraph-2k, Innsbruck, Austria) to measure mitochondrial respiration in *Drosophila* larvae was used as described before [23]. Ten third-instar larvae were rapidly dissected, removing the tracheal system and the rest of the organs under a microscope, and mechanically homogenized in Miro 6 Buffer (20 mM Hepes, 110 mM sucrose, 10 mM KH_2_PO_4_, 20 mM taurine, 60 mM lactobionic acid, 3 mM MgCl_2_, 0.5 EGTA, pH 7.1, 1 mg/mL fatty acid-free BSA, catalase 280 U/mL) [96], then immediately loaded into an Oroboros Oxygraph-2K chamber filled with Miro 6 buffer equilibrated at 25 °C.

Oxygen consumption rates were measured before and after the addition of the following sequence of substrates and specific inhibitors: (1) A volume of 2.5 mM pyruvate, 1 mM malate in flies, and a mixture of 2.5 mM pyruvate, 10 mM glutamate, and 1 mM malate in human cells (CI leak), followed by 2.5 mM ADP to determine complex I-driven phosphorylating respiration (CI OXPHOS). (2) A volume of 5 mM succinate to determine the phosphorylating respiration driven by complex I and II (CI + II OXPHOS). (3) Titration of the mitochondrial uncoupler CCCP concentrations to reach the maximal, uncoupled respiration (CI + II electron transfer system, ETS). (4) A volume of 200 nM rotenone to fully inhibit complex I-driven respiration and measure complex II-driven uncoupled respiration (CII electron transfer system, CII ETS). (5) A volume of 0.5 µM Antimycin A to block mitochondrial respiration at the level of complex III. (6) A volume of 2 mM ascorbate and 0.5 mM TMPD to measure cytochrome c oxidase (CIV)- driven respiration. Residual oxygen consumption was measured by adding Sodium Azide that blocks cytochrome c oxidase.

ATP levels were measured from third-instar larvae lysates using the ADP/ATP Ratio Bioluminescence Assay Kit, ApoSENSOR (Biovision, Milpitas, CA, USA).

### 4.7. RNA Extraction and RT-qPCR

Total RNA was extracted from third-instar larvae using Trizol reagent (Invitrogen, Carlsbad, CA, USA) according to the manufacturer’s instructions. cDNA was prepared using M-MLV Reverse Transcriptase (200 U/uL, Invitrogen, Waltham, MA, USA, cat. 28025013) and random primers (Promega, Madison, WI, USA). qPCR was performed on a Light Cycler 96 (Roche, Switzerland) with the SYBR Green-based detection system (Roche, Basel, Switzerland, cat. 04887352001) with primers of our genes of interest: *RLP32* forward AGCATACAGGCCCAAGATCG; *RLP32* reverse TGTTGTCGATACCCTTGGGC; *SLR1* forward ACTGCAACTGACAGATACACTG; and *SLR1* reverse CCTCCCGGTTATGGTTGAGC. Two technical replicates for each biological replicate were assessed. SLR1 (PGC1α) levels were normalized to RPL32 using the comparative ΔΔCT method.

### 4.8. Statistical Analysis

All data were analyzed using appropriate statistical methods to ensure the validity and reliability of the results. For the locomotor behavior experiments (Figure 1), data were quantified automatically with EthoVision, measuring the distance and speed of larvae. Each condition included a minimum of 15 individual larvae, with data presented as dot plots and error bars representing the standard error of the mean (SEM). Statistical significance was determined using 2-way ANOVA with multiple comparisons (* *p* < 0.05, ** *p* < 0.01, *** *p* < 0.001).

For the analysis of NMJ synaptic transmission (Figure 2), both spontaneous and evoked recordings were quantified. Each dot represents the measurement from a single NMJ, with a minimum of 7 independent larvae NMJs per condition. Data were presented as dot plots, with error bars showing SEM. Statistical significance was assessed using 2-way ANOVA with multiple comparisons (* *p* < 0.05, ** *p* < 0.01, *** *p* < 0.001).

Mitochondrial activity was assessed by quantifying oxygen consumption normalized to protein content (Figure 3A) and ATP production levels (Figure 3B). Each condition included at least 4 independent experiments per genotype (each with 3 NMJs). Data were expressed as mean ± SEM. Oxygen consumption data were analyzed using multiple *t*-tests corrected for multiple comparisons with the Sidak–Bonferroni method (** *p* < 0.01). ATP production levels were compared using *t*-tests (** *p* < 0.01).

Mitochondrial populations and distributions were quantified using mito-GFP expression and anti-HRP staining (Figure 4). Each dot represents a single NMJ, with at least 15 independent NMJs analyzed per condition. Data were shown as dot plots ± SEM and statistical significance was determined by 2-way ANOVA with multiple comparisons (* *p* < 0.05).

For the modulation of mitochondrial biogenesis and its effect on motor behavior (Figure 5), locomotor behavior was quantified by measuring the distance moved by larvae in 1 min and their crawling speed. Each condition included a minimum of 7 single larvae. Data were shown as dot plots with error bars representing SEM. Statistical significance was determined using the Kruskal–Wallis test followed by Dunn’s multiple comparisons test (** *p* < 0.01).

These comprehensive statistical analyses ensure the robustness and reliability of our findings across various experimental conditions.

## Data Availability

Data supporting the reported results can be obtained upon request from the corresponding authors.
